# The complete chloroplast genome sequence of *Achimenes cettoana* (Gesneriaceae)

**DOI:** 10.1080/23802359.2020.1847607

**Published:** 2021-03-17

**Authors:** Shu Li, Rui Liao, Zi-Bing Xin, Zhang-Jie Huang, Stephen Maciejewski, Fang Wen

**Affiliations:** a Guangxi Key Laboratory of Plant Conservation and Restoration Ecology in Karst Terrain, Guangxi Institute of Botany, Guangxi Zhuang Autonomous Region and Chinese Academy of Sciences, Guilin, PR China; b Gesneriad Conservation Center of China, Guilin Botanical Garden, Chinese Academy of Sciences, Guilin, PR China; c Hubei Provincial Key Laboratory for Protection and Application of Special Plant Germplasm in Wuling Area of China, Key Laboratory of State Ethnic Affairs Commission for Biological Technology, College of Life Science, South-Central University for Nationalities, Wuhan, PR China; d The Gesneriad Society, Philadelphia, PA, USA

**Keywords:** *Achimenes*, chloroplast genome, phylogenetics

## Abstract

*Achimenes* Pers. is well known for its floral diversity and widely used in horticulture, but its phylogenetic position is still poorly understood. And most research about the complete chloroplast genome sequence focused on the Old World species; therefore, we think it is necessary to examine taxa of the New World in more detail. This study determined the complete chloroplast genome of *Achimenes cettoana* H.E. Moore. The cp genome was 153,011 bp in a total length containing two inverted repeats (IRs) of 25,162 bp separated by a large single-copy (LSC) region of 84,669 bp and a small single-copy (SSC) region of 18,018 bp. The whole cp genome of *A. cettoana* contains 112 genes, including 79 protein-coding genes, 29 *tRNA* genes, and four *rRNA* genes. This plastid genome is firstly reported in the New World Gesneriaceae, which will be a valuable resource for future studies on breeding, conservation genetics, and phylogeny of Gesneriaceae.

Based on molecular phylogenetic studies, the first detailed and overall classification of Gesneriaceae was made by Weber et al. (2013), three subfamilies were recognized: Sanangooideae (monospecific with *Sanango racemosum* G.S. Bunting & J.A. Duke), Gesnerioideae, and Didymocarpoideae. Both Subfam. Sanangooideae and Subfam. Gesnerioideae are distributed in the New World (*Titanotrichum* Soler. excluded), and Subfam. Didymocarpoideae is distributed in the Old World. *Achimenes* Pers. (belonging to the Subfam. Gesnerioideae) is an herbaceous genus of about 24 species (IPNI 2020; Tropicos 2020), distributed from southern Mexico through Central America (Roa et al. 2002; Roalson and Roberts 2016).

The DNA sequence of the chloroplast genome can be used as a valuable resource for future studies on breeding, conservation genetics and phylogeny. Within Gesneriaceae, all chloroplast genomes reported belong to Subfam. Didymocarpoideae and no complete chloroplast sequence for the New World Gesneriaceae have been published to date.

The members of the Gesneriad Society collected seeds of *Achimenes cettoana*, which grows on rocks in the stream of a shaded ravine, from Chiapas, Mexico (GPS: 16.74 N, 92.69 W). Then seeds were shared to GCCC, and were being cultivated in the nursery of GCCC since 2015. *Achimenes cettoana* is a compact plant and adapts well to cultivation (Moore 1961), especially for a windowsill or under artificial light. The voucher specimen (HZJ001) was deposited at the Herbarium of Guangxi Institute of Botany, CAS (IBK). The chloroplast DNA of *A. cettoana* was extracted by the CTAB method (Doyle and Doyle 1987) and sequenced using the Illumina Hiseq 4000 sequencing platform (Majorbio Company (http://www.majorbio.com/, China). We used Map to Reference function in Geneious R11 (Kearse et al. 2012) to exclude nuclear and mitochondrial reads using the plastid genome of *Petrocodon jingxiensis* (GenBank-MK887172) as reference. Putative chloroplast reads were used for de novo assembling construction using Geneious R11. Generated contigs were concatenated into larger ones using the Repeat Finder function in Geneious R11. The original data were again mapped to the larger contigs to extend their boundaries until all contigs were able to concatenate to one contig. The inverted repeat (IR) region was determined using the Repeat Finder function in Geneious R11 and was inverted and copied to obtain the complete chloroplast sequence. DOGMA program was used for annotation of the complete chloroplast genome (Wyman et al. 2004) and the annotation was corrected with Geneious R11 (Kearse et al. 2012). The complete chloroplast genome of *A. cettoana* was 153,011 bp in length (MT627324), and the GC content was 38.2%. Large single-copy (LSC) and small single-copy (SSC) contained 84,669 bp and 18,018 bp, while IRs were 25,162 bp in length. The plastid genome included 79 protein-coding genes, 29 *tRNA* genes, and four *rRNA* genes.

To analyze the phylogenetic position of *A. cettoana*, the concatenated sequences of protein-coding genes of *A. cettoana* and 11 other species from Gesneriaceae were aligned using MAFFT version 7.307 (Katoh and Standley 2013) and 3 species from Labiatae and Acanthaceae were selected as outgroup. A phylogenetic tree (Figure 1) was constructed with the maximum likelihood (ML) method and the TVM + F + R2 evolutionary model using RAxML (Stamatakis 2014). The result was congruent with previous studies that established that *Achimenes* is a member of the New World Gesneriaceae (Clark et al. 2012; Weber et al. 2013). The topology of this molecular tree supports the division of this subfamily and shows that *A. cettoana* is a representative of Subtr. Gloxiniinae and is a sister group of Gesneriinae (Clark et al. 2012). The newly reported chloroplast genome of *A. cettoana* allows developing markers for further studies on resolving the relationship within the Subfam. Gesnerioideae and particularly, the genus *Achimenes*.

**Figure 1.**
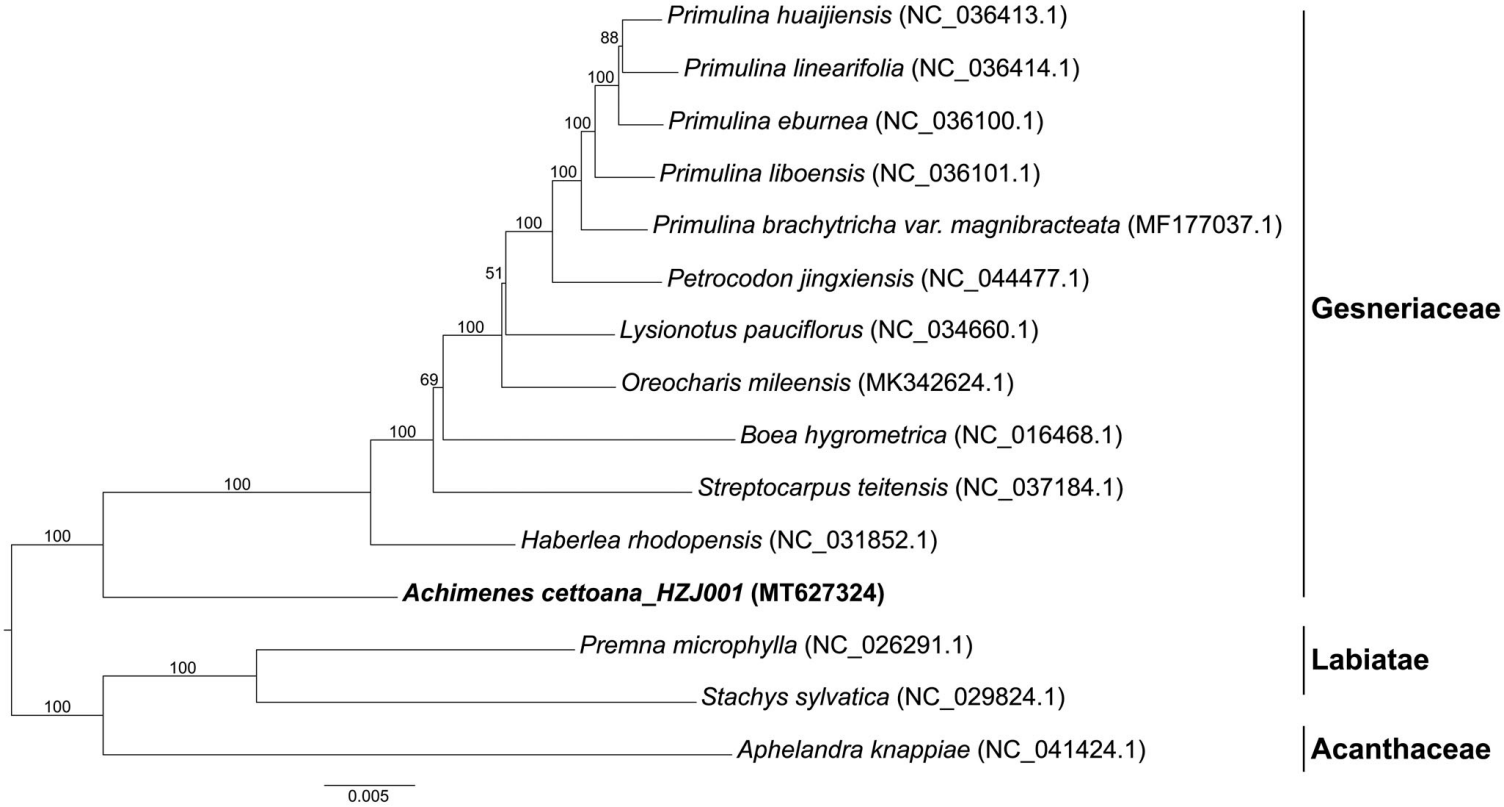
Phylogenetic tree reconstructed by maximum likelihood (ML) analysis based on concatenated sequences of protein-coding genes from 15 taxa of Lamiales, numbers upon branches are assessed by ML bootstrap.

## Data Availability

The data that support the findings of this study are openly available in GenBank of NCBI at https://www.ncbi.nlm.nih.gov, reference number MT627324.
